# Inheritance and Resistance Mechanisms of Field-Evolved Resistance to Pyrethroids in a Fall Armyworm (*Spodoptera frugiperda* J.E. Smith) (Lepidoptera: Noctuidae) Strain from Puerto Rico

**DOI:** 10.3390/insects15120912

**Published:** 2024-11-21

**Authors:** Omar Alejandro Posos-Parra, Barry R. Pittendrigh, John C. Wise, Christina DiFonzo, Eric Patterson, David Mota-Sanchez

**Affiliations:** 1 Department of Entomology, Michigan State University, East Lansing, MI 48824, USA; posospar@msu.edu (O.A.P.-P.); wisejohn@msu.edu (J.C.W.); difonzo@msu.edu (C.D.); 2 Department of Entomology, Purdue University, West Lafayette, IN 47907, USA; pittendr@purdue.edu; 3 Department of Plant, Soil, and Microbial Sciences, Michigan State University, East Lansing, MI 48824, USA; patte543@msu.edu

**Keywords:** pyrethroids, FAW, esfenvalerate, deltamethrin, resistance, inheritance, practical resistance, Puerto Rico

## Abstract

This study examines resistance inheritance to the pyrethroid insecticides esfenvalerate and deltamethrin in a Puerto Rican strain of fall armyworm (FAW), *Spodoptera frugiperda*, a major global pest of corn. The resistant strain (PPR) showed significantly higher resistance compared to a susceptible strain (SUS), with a 62-fold X-linked and 15-fold autosomal-linked resistance ratio (RR_50_) for esfenvalerate and deltamethrin, respectively. Resistance was incompletely dominant for both insecticides. Synergist bioassays revealed that detoxification enzymes play a key role in resistance, with PPR exhibiting increased toxicity across all tested synergists, especially with a 12-fold increase when all were combined. Deltamethrin assays confirmed the importance of these enzymes, with a 17-fold increase in PPR toxicity when combined with esterase inhibitors. These findings highlight the complexity of pyrethroid resistance, involving multiple non-target site mechanisms, and suggest that heterozygous individuals could survive in treated crops due to incomplete dominance. The results emphasize the need for diversified pest management strategies, including insecticide rotation, to effectively control FAW populations.

## Simple Summary

Fall armyworm (FAW) is a pest that severely devastates corn and other crops in most of the continents. It has developed resistance to numerous synthetic insecticides, rendering its management increasingly challenging. This study investigates the resistance of FAW in Puerto Rico to the pyrethroids esfenvalerate and deltamethrin, which have become less effective, likely due to the pest’s ability to develop resistance. The research findings indicate that FAW had developed high levels of field-evolved resistance to pyrethroids, and the resistance was partially inherited and X-linked. Through the utilization of enzyme inhibitors of P450s, esterases, GSHs, and ABC transporters, it was determined that these enzymes play a crucial role in FAW’s defense against pyrethroids. These findings have global implications due to the invasion of FAW to Africa, Asia, Oceania, and Europe, where pyrethroids are commonly used to manage FAW. There is a critical need for strategies in FAW management, such as the rotation of different insecticides or the integration of alternative pest control methods, particularly in regions similar to Puerto Rico, where FAW pressure is very high, to ensure the stability of global food production, especially seed production, which is vital for food security globally and at the local scale.

## 1. Introduction

The fall armyworm (FAW), *Spodoptera frugiperda* (J.E. Smith) (Lepidoptera: Noctuidae), is one of the most economically significant pests of the twenty-first century, causing extensive damage to corn and various other crops. FAW possesses several formidable traits that contribute to its prominent pest status, including a high reproductive rate, multiple generations per year, lack of diapause, rapid adaptation to new environments, and a broad host range encompassing hundreds of species [1,2,3,4,5]. Additionally, FAW has evolved resistance to a wide variety of insecticides [6]. As of now, there are 194 reported cases of FAW resistance to 45 different active ingredients, spanning eight modes of action [7].

FAW is native to Latin America, the Caribbean islands, and the southernmost US, although it migrates annually as far north as the US Corn Belt and Canada [8,9]. However, in 2016, there was a pivotal shift in the FAW’s distribution and economic impact [10]. For the first time, infestations were reported in Africa [3,11], eventually expanding across the continent [12,13,14], then to Asia (Republic of Korea, India, China, Japan, Pakistan, and Vietnam) and Oceania (Australia) [15,16,17,18,19,20]. Most recently, it was found in Saudi Arabia, the Canary Islands, and Turkey.

FAW feeding can result in yield reductions of over 60% in corn, a critical crop for global food security [21,22]. It also poses an over-looked, but significant, challenge for the seed industry. Puerto Rico plays an essential role in agricultural seed production as both a research and bulk-seed production hub. Given its tropical climate, Puerto Rico can support up to four corn crops per season. Remarkably, approximately 85% of all certified field crop seeds used for global food consumption pass some stage of development in Puerto Rico’s fields and nurseries [23]. At the same time, the tropical conditions create an ideal environment for FAW, resulting in up to ten generations of persistent high pressure per year [6,24,25]. Since there is low tolerance for kernel damage in seedcorn production, the industry resorts to intensive pesticide usage, with up to thirty applications per season of products from at least nine modes of action [24]. Unfortunately, sustained pest pressure and extensive insecticide use have led to the evolution of broad-spectrum pesticide resistance in FAW populations in Puerto Rico. Notably, resistance has been observed to a range of synthetic insecticides, including pyrethroids [6,24,25].

Pyrethroids (Group 3, IRAC) have been integral components of integrated pest management (IPM) strategies since the 1970s, favored globally because of their lower mammalian toxicity compared to older conventional pesticide groups. Pyrethroids have an established safety profile because insects are intrinsically more susceptible than mammals [26]. However, recent publications have documented the potential risk of pyrethroids for human health [27]. Their activity arises from their interference with neurotransmission at insect voltage-gated Na+ channel recognition sites, blocking Na+ transport, extending the Na+ current duration during depolarization, and eliciting a lingering slow current (“tail current”). This chain of events culminates in instant paralysis [28,29,30,31].

Pyrethroids are classified in two distinct categories, Type I and Type II, based on chemical structure, sensory neuron activity, and the poisoning symptoms [31,32,33,34]. Type I pyrethroids lack an α-cyano group at the phenylbenzyl alcohol position, while Type II pyrethroids possess this group. Functionally, Type I pyrethroids prompt repetitive discharges in sensory neurons, without initiating neurotransmitter release. In contrast, Type II pyrethroids do not produce these repetitive discharges, leading to an extended tail current decay. Furthermore, Type I pyrethroids exhibit a negative temperature–toxicity correlation, with higher toxicity at lower temperature; Type II pyrethroids display the opposite trend [35,36].

True resistance only occurs when a structural genetic change that is heritable takes place [37]. This concept is exemplified by the resistance to pyrethroids in the fall armyworm (FAW), which has evolved across multiple regions (Table 1). In Puerto Rico, over a decade of continuous reliance on pyrethroids, particularly esfenvalerate and deltamethrin (both Type II pyrethroids), has driven the development of practical resistance, significantly reducing their effectiveness in controlling FAW populations in cornfields. The intensity of pesticide application creates substantial genetic selection pressure at the population level, fostering conditions where resistance can emerge and persist [6,24,38,39].

Research into the inheritance of resistance, supported by physiological and biochemical tests, has provided valuable insights into the microevolutionary processes involved, revealing unexpected complexities in arthropod resistance mechanisms [39]. This study elucidates the inheritance patterns and metabolic mechanisms associated with esfenvalerate and deltamethrin resistance in FAW populations in Puerto Rico. By shedding light on the genetic basis of resistance and understanding the role of synergists, this study contributes to a deeper understanding of FAW resistance and offers insights for the development of effective FAW management strategies in seed production in Puerto Rico and elsewhere.

## 2. Materials and Methods

### 2.1. Insect Populations

A FAW field strain from Ponce, Puerto Rico (PPR), originated from a collection of larvae from an infested cornfield. The larvae were shipped in cups with diet to Michigan State University, where they were identified [54,55,56,57,58] and separated to initiate the rearing process. A susceptible FAW colony (SUS) was provided by Bayer USA from their research facilities in Memphis, Tennessee. We have utilized this susceptible strain continuously for over eight years.

Throughout all larval cycles, colonies were maintained in 60 mL cups with 10 mL of artificial FAW diet (Southland Products Inc., Lake Village, AR, USA). After pupation, thirty reciprocal pairs were placed in 5 L paper brown bags for mating, and the bags were placed inside mesh cages. To feed adults, 10 ml cups with cotton balls impregnated with a liquid solution of Gatorade^®^ lime or orange flavor were placed in the bags. The bags were checked twice weekly for food maintenance and oviposition of egg masses on the bag surface. Egg masses were placed in cups with an artificial diet until they hatched; then, first instars were placed into individual cups using a paintbrush to avoid damage. Temperature and photoperiod conditions of 26 ± 2 °C and 16:10 h (L:D), respectively, were used for both the PPR and SUS colonies. All insects were checked daily to confirm the correct and healthy development of both strains [6].

### 2.2. Chemicals and Insecticides

For all bioassays, commercial formulations of the pyrethroids esfenvalerate (Asana XL EC, 8.4%, 79 g a.i./L, Valent USA Corporation, Walnut Creek, CA, USA) and deltamethrin (Battalion™ 0.2 EC, 2.86%, 23.96 g a.i./L, Arysta LifeScience, Cary, NC, USA) were used. Analytic-grade synergist compounds and organic solvents were purchased from (Sigma-Aldrich, St. Louis, MO, USA).

### 2.3. Pyrethroid Bioassays

Concentration–response bioassays of esfenvalerate and deltamethrin were carried out via diet overlay bioassays in 24-well trays (ProCell, Alkali Scientific Inc., Fort Lauderdale, FL, USA). Each well was filled with 1 mL of the FAW artificial diet (Southland Products Inc., Lake Village, AR, USA) treated with 30 μL of each insecticide solution to cover a surface area of 2.0 cm^2^. The concentrations of the insecticides varied to cover a range of mortality from 5% to 95%, with five to nine concentrations per insecticide and four replicates per concentration. The control treatment consisted of 30 μL of distilled water with a surfactant at 0.05% (*v*/*v*).

Each replicate included 12 wells, with one third instar per well. After the application of the solution, the trays were left to dry for approximately one hour before introducing FAW larvae to the treated surface. After four days, mortality was recorded. Individuals showing acute intoxication symptoms (necrotic tissue, slow movement, or interrupted molting) or those that did not respond to stimulation with a small paintbrush or forceps were considered dead.

### 2.4. Inheritance of Resistance

Using sexual dimorphism, we collected and separated pupae from both populations into female and male groups [59]. Then, reciprocal crosses were made using thirty pairs of adult FAWs per each F_1_ crosses were defined as H1 (♂ SUS × ♀ PPR) and H2 (♀ SUS × ♂ PPR).

To evaluate the dominance of resistance, larvae from the reciprocal crosses were subjected to the same susceptibility bioassays used for the SUS and PPR populations in Section 2.3. The degree of dominance was estimated using the equation from Bourguet et al. [60].

D_M_ = (M_RS_ − M_SS_)/(M_RR_ − M_SS_) (1)

where M_SS_, M_RS_, and M_RR_ were the mortalities expressed in µg/cm^2^ of the SUS, reciprocal crosses (H1 or H2), and PPR population, respectively, at different pyrethroid concentrations. D_M_ values close to 1 were considered completely dominant inheritance, whereas values close to 0 were deemed completely recessive inheritance. To understand the trend of resistance dominance versus concentration, a range of concentrations covering both reciprocal crosses was established, where mortalities were found (2–98%). Data were further analyzed using Stone’s equation [61] to determine the degree of dominance at the LC_50_.

D = (2Y_2_ − Y_1_ − Y_3_)/(Y_1_ − Y_3_) (2)

where Y_1_ and Y_2_ represent the log10 LC_50_ values for the reciprocal crosses (H1 or H2 heterozygotes) and Y_3_ corresponds to the log10 LC_50_ for the parental populations (PPR and SUS), respectively. D values were interpreted as follows: −1, completely recessive; −1 < D < 0, incompletely recessive; 0 < D < 1, incompletely dominant; and D = 1, completely dominant.

### 2.5. Synergist Bioassays

Bioassays combining pyrethroids and synergists were conducted to investigate the function of detoxification enzymes. The following compounds were tested: (1) the cytochrome P450 inhibitor piperonyl butoxide (PBO 91.3%, SynerProTM Control Solutions Inc. Pasadena, TX, USA); (2) the esterase inhibitor S,S,S-tributyl phosphorotrithioate (DEF 98.1%, Sigma-Aldrich, Saint Louis, Missouri, USA); (3) the glutathione S-transferase inhibitor diethyl maleate (DEM 97%, Sigma-Aldrich, St. Louis, MO, USA); and (4) the ABC transporter inhibitor (±)-verapamil hydrochloride (VER 99%, Sigma-Aldrich, St. Louis, MO, USA). To determine the synergist concentration to use in the combined assay, separate diet overlay bioassays were performed to find the maximum non-lethal concentration for each synergist alone in the third instar. The highest concentrations of each compound that did not cause mortality or loss of fitness in the larvae 96 h after application were 4.5 μg/cm^2^, 1.5 μg/cm^2^, 0.45 μg/cm^2^, and 0.45 μg/cm^2^ for PBO, DEF, DEM, and VER, respectively.

Pyrethroid + synergist bioassays were conducted using the same procedure as the pyrethroid bioassays in Section 2.3, with mortality rates assessed at four days after application. Mortality probit analyses and data plotting were also estimated, with synergist ratios (SR_50_ and SR_90_) calculated by dividing the LC_50_ and LC_90_ values of the pyrethroid alone by the LC_50_ and LC_90_ values of the pyrethroid with synergist concentration. Each set included four replicates, and each replicate consisted of 12 wells with five to seven concentrations each. Every well contained a single third-instar larva, resulting in a total of 48 wells per bioassay.

### 2.6. Statistical Analysis

Probit analysis [62] was used to analyze bioassay results using the PROC PROBIT procedure from SAS version 9.4 [63]. This analysis estimated the slope values, standard error, lethal concentrations at 50% (LC_50_) and 90% (LC_90_), fiducial limits (95%), and χ^2^ for each population. The resistance ratios (RR_50_ and RR_90_) were obtained by dividing the LC_50_ and LC_90_ values of the PPR population by those of the susceptible population (SUS). Mortality data were adjusted using Abbott’s equation [64]. The log concentration detoxification responses of both populations were compared using parallelism and equality tests (*p* < 0.05) with PoloJR [65]. Graphs and log concentration responses were generated using Prism GraphPad Software version 10 [66].

## 3. Results

### 3.1. Bioassays and Inheritance of Resistance

**Figure 1:**
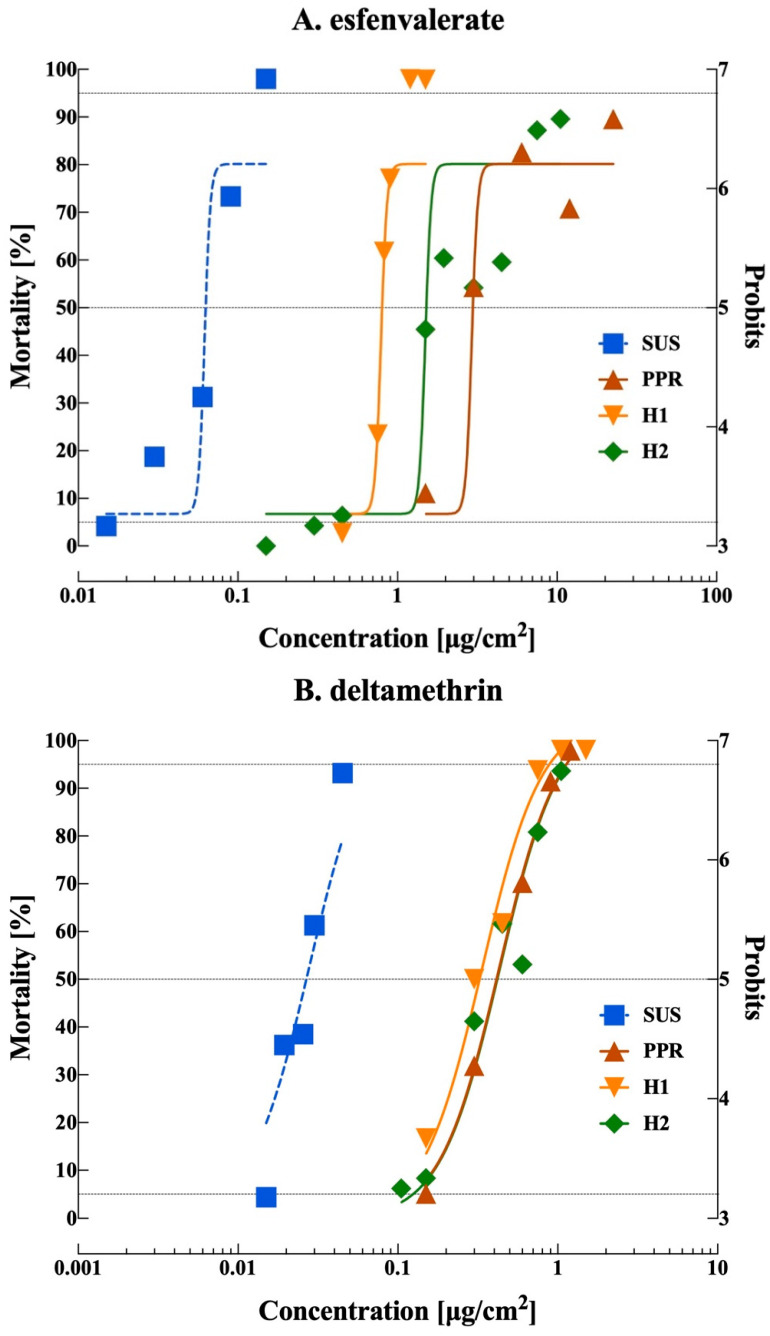
Mortality response of fall armyworm from a susceptible lab colony (SUS), a field collection from Ponce Puerto Rico (PPR), and their F_1_ reciprocal crosses (H1, ♂ SUS × ♀ PPR) + (H2, ♀ SUS × ♂ PPR) to esfenvalerate and deltamethrin.

The PPR population exhibited a 62-fold RR_50_ for esfenvalerate and 15-fold RR_50_ for deltamethrin compared to the SUS strain. For esfenvalerate, there was no overlap in confidence intervals for LC_50_ (95% CI) between the H1 and H2 populations (Table 2). The absence of overlapping LC_50_ values suggests an X-linked inheritance of resistance. For deltamethrin, the LC_50_ (95% CI) did overlap, suggesting an autosomal inheritance of resistance (Figure 1). Compared to the SUS strain, the RR_50_ values for esfenvalerate were 13-fold and 34-fold for H1 and H2, and they were 7-fold and 15-fold for H1 and H2 for deltamethrin. A comparison of detoxification using parallelism (χ^2^ = 157, d.f. = 3, *p* < 0.05) and equality (χ^2^ = 152.7, d.f. = 4, *p* < 0.05) revealed a unique response for each pyrethroid in the field-evolved strain from PPR.

Based on Stone’s method [61], the degree of dominance D at the LC_50_ for esfenvalerate was 0.249 and 0.741 and for deltamethrin 0.791 and 0.986 for H1 and H2, respectively. These results suggest that the resistance was incompletely dominant for H1 and H2 strains for both active ingredients.

The degree of dominance calculated from the equation in Bourguet et al. [60] for both active ingredients followed a similar trend between crosses. In the H1 progeny (♂ SUS × ♀ PPR), the response to esfenvalerate shows an initial increase in dominance with concentration, reaching its peak at D_M_ values just below 0.75 (0.10 μg/cm^2^). Beyond this concentration, the dominance level decreases, stabilizing around D_M_ = 0.5, indicating a shift from complete to incomplete dominance. For deltamethrin in the H1 progeny, a different pattern emerges, with dominance levels approaching complete dominance (D_M_ close to 1.0) across all concentrations, except at the highest tested concentration, where it shows a slight reduction. In the H2 progeny (♀ SUS × ♂ PPR), the dominance pattern for both esfenvalerate and deltamethrin is more uniform. For deltamethrin, the D_M_ values are consistently close to 1.0 across all concentrations, indicating a strong, almost completely dominant inheritance. Similarly, for esfenvalerate in H2, dominance also approaches complete dominance (D_M_ close to 1.0) across all concentrations, except at the highest tested dose, where it deviates slightly.

**Figure 2:**
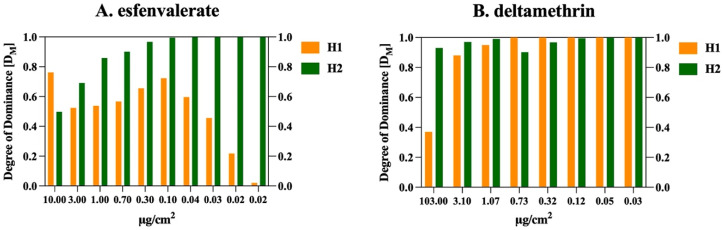
Dominance degree [D_M_] of resistance to esfenvalerate (**A**) and deltamethrin (**B**) in F_1_ reciprocal crosses (H1, ♂ SUS × ♀ PPR) + (H2, ♀ SUS × ♂ PPR) of FAW.

Thus, for deltamethrin, both H1 and H2 progeny exhibit a trend towards complete dominance across the range of concentrations, with a minor exception at the highest dose. For esfenvalerate, while H1 progeny show a trend towards incomplete dominance at higher concentrations, H2 progeny maintain a pattern of complete dominance across most concentrations (Figure 2).

### 3.2. Synergism Bioassay

The results of the esfenvalerate synergist bioassays demonstrated that both SUS and PPR strains used a detoxification enzyme system to metabolize pyrethroids. In the SUS strain, the P450s inhibitor (PBO) was the most effective, resulting in a 4.6-fold increase in synergism (SR_50_), followed by the esterase inhibitor (DEF), with a 1.4-fold increase. The glutathione S-transferase inhibitor (DEM) and ABC transporter inhibitor (VER) showed no synergism (<1-fold).

In the PPR strain, all four synergists caused synergism combined with esfenvalerate, indicating the presence of detoxification enzymes. The PBO bioassays showed a 3.5-fold increase in synergism, whereas the VER bioassays showed a 4.7-fold increase. The DEF and DEM bioassays showed a similar trend, with the highest increase in synergism of almost 8-fold. When all synergists were combined in an additional bioassay, there was an accumulative effect, resulting in a 12-fold increase in SR_50_ in the field strain (Table 3).

The results of the deltamethrin synergist bioassays indicated similar detoxification enzyme roles. In the SUS strain, the most significant synergism was found in bioassays with the presence of PBO, with a SR_50_ of 3-fold, followed by DEF bioassays with 1.3-fold. In the DEM and VER bioassays, a minor antagonism was found in the presence of such synergists (<1-fold). However, in the PPR population, higher synergism was found in DEF, with a 17-fold increase, followed by VER with a 4-fold increase, and PBO and DEM bioassays with a 2-fold increase (Table 4). These findings suggest that both strains (SUS and PPR) have different detoxification mechanisms for esfenvalerate and deltamethrin and that the presence of detoxification enzymes can significantly affect the toxicity of both pyrethroids.

## 4. Discussion

In this study, we investigated the inheritance of resistance to two pyrethroids in a field-evolved resistant FAW population from Puerto Rico and the contribution of detoxifying enzymes to pesticide resistance. The resistance of FAW to esfenvalerate (62-fold) and deltamethrin (15-fold) resulted in “practical resistance”. This is the first report of resistance to esfenvalerate in Puerto Rico. Prior studies on field-evolved resistance to pyrethroids in an FAW strain from the same geographical location in indicated resistance to permethrin, deltamethrin, and zeta-cypermethrin [6]. The continuous use of esfenvalerate to manage FAW over the last decade in all cropping seasons has resulted in high levels of resistance [67]. Practical resistance to pyrethroids in the field has been reported, prompting a re-formulation of strategies to rotate action modes and explore innovative IPM programs that utilize all accessible resources.

Reciprocal crosses revealed a noteworthy pattern: heterozygous individuals exhibited an incomplete dominant response to esfenvalerate and deltamethrin, as shown in Figure 1A,B. These data suggest that heterozygous larvae can tolerate concentrations akin to their homozygous resistant counterparts, subsequently increasing the gene frequency in field populations (Figure 2). This observed resistance becomes even more pronounced as pesticide residue decays; heterozygous resistant larvae seem to endure and thrive, leading to a swift evolution of resistance in the field. The dynamics of this resistance pattern are especially significant when considering the ubiquity of pyrethroids; they stand out not only for their cost-effectiveness, especially when compared to newer materials such as diamides [16], but also their frequent integration into IPM programs [32,68]. This widespread reliance could intensify selection pressure, resulting in individuals experiencing indirect exposure to sublethal doses at various stages.

With its tropical conditions, Puerto Rico presents a unique environment in which continuous oviposition leads to an ever-present cycle of six larval stages and overlapping FAW generations. Within this context, a compelling hypothesis emerges: the pesticide resistance landscape in Puerto Rico might align with a broader theme of intra-island variation in susceptibility. This pattern implies that the island’s gene flow might be insufficient to balance out differences in insecticide susceptibility, a phenomenon echoed in species such as the green aphis (*Aphis gossypii*) [69], whitefly (*Bemisia tabaci*) [70], and diamondback moth (*Plutella xylostella*) [71], predominantly from Hawaii. However, in order to test this hypothesis, it would be imperative to carry out bioassays involving diverse FAW strains from Puerto Rico.

Resistance to pyrethroids (esfenvalerate and deltamethrin), which is inherited in an incompletely dominant manner, is commonly observed in several species. For instance, a similar inheritance trend has been found in species closely related to the diamondback moth [72], the predator lady beetle (*Eriopis connexa*) to deltamethrin [73], the cotton bollworm (*Helicoverpa armigera*) to cypermethrin [74] and fenvalerate [75,76], the soybean looper (*Chrysodeixis includens*) to lambda-cyhalothrin [77], the two spotted spider mite (*Tetranychus urticae*) to lambda-cyhalothrin as well [78], the tobacco budworm (*Heliothis virescens*) to permethrin [79], and the horn fly (*Haematobia irritans*) to cypermethrin.

The inheritance of resistance indicated a sex-linked inheritance pattern for resistance to esfenvalerate in the FAW population from Ponce, Puerto Rico, suggesting that males predominantly transmit this resistance. This type of inheritance pattern is rare in FAW, since we have not found a publication indicating this pattern of resistance. A previous example included a field-evolved resistant strain of the convergent lady beetle (*Hippodamia convergens*) from Georgia, USA, which demonstrated a sex-linked recessive inheritance pattern for resistance to lambda-cyhalothrin [80]. A similar pattern was observed in the two-spotted spider mite from Antalya, Turkey [78]. More recently, evidence of a sex-linked flubendiamide resistance pattern has been observed in a population from the same geographic area [81].

In contrast, resistance to deltamethrin in FAW appears to be autosomally inherited, a finding corroborated by studies on a lab-selected FAW population resistant to lambda-cyhalothrin from Guaría, Sao Paulo [44]. Previous reports also identified autosomal resistance traits in FAW from other regions of the Americas to a range of other insecticides, including carbamates (carbaryl) [82], organophosphates (chlorpyrifos) [83], pyrethroids (lambda-cyhalothrin) [44], nicotinic acetylcholine receptor (nAChR) allosteric modulators, spinosyns (spinosad and spinetoram) [84,85], glutamate-gated chloride channel (GluCl) allosteric modulators, avermectins and milbemycins (emamectin benzoate) [86], and inhibitors of chitin biosynthesis, benzoylureas (novaluron and teflubenzuron) [87,88]. Autosomal inheritance of deltamethrin resistance has also been documented in other species. These include the diamondback moth [89], house fly (*Musca domestica*) [90], common lacewing (*Chrysoperla carnea*) [91], tobacco cutworm (*Spodoptera litura*) [89,92], and codling moth (*Cydia pomonella*) [93].

The use of synergists plus pyrethroids indicated the crucial role of detoxification enzymes in resistance mechanisms. This research particularly underscores the importance of a variety of detoxification enzymes when studying esfenvalerate resistance. Enzymes, such as P450s, esterases, glutathione S-transferase enzymes, and ABC transporters, are integral components of the resistance mechanism. Similar detoxification mechanisms have been observed in other instances, such as the soybean aphid’s response to lambda-cyhalothrin [94], the western flower trips (*Frankliniella occidentalis*) to tau-fluvalinate [95], and the cotton bollworm to fenvalerate [76] and cypermethrin [96,97].

Resistance to pyrethroids in FAW is characterized by metabolic enzymes and mutations at the target site [37,45]. Owing to their unique chemical structure, pyrethroids undergo phase I detoxification reactions, such as hydrolysis [32]. Enzymes such as cytochrome P450s and esterases play critical roles in detoxification in insects. The use of inhibitors of these enzymes, such as PBO for P450s and DEF for esterases, enhances the toxicity of pyrethroids, thereby increasing their susceptibility [98]. Some of the resistance mechanisms may be triggered by genetic mutations.

The use of synergists with esfenvalerate suggested a high involvement of P450s, esterases, glutathione S-transferases, and ABC transporters in the suppression of resistance, with the combination of all synergists being the most promising factor of resistance (Table 3). For deltamethrin, synergist bioassays indicated reduced involvement of P450s, glutathione S-transferases, and ABC transporters but also a significant presence of esterases in the detoxification process. Considering the complexity of these biochemical interactions, it is plausible to hypothesize that the resistance observed in response to both pyrethroids may be polygenic in nature. However, this study’s scope did not extend to performing backcross tests with the field-derived PPR colony, representing a key limitation. Future research should address this gap to uncover a more detailed genetic foundation underlying these resistance patterns, potentially clarifying the polygenic factors involved.

Furthermore, the metabolic effort required to sustain a high level of metabolic defense likely imposes a fitness cost. Such a cost could be disadvantageous in the absence of selective pressure, suggesting that resistance may wane when artificial selection is no longer applied, thereby influencing population dynamics and resistance sustainability.

More detailed molecular analysis is needed to elucidate other roles in the resistance mechanism of FAW from Puerto Rico [99], since the synergists in mix with pyrethroids were not able to fully suppress the resistance levels in this study. Mutations at the target site have been extensively studied because of the mode of action of pyrethroids in voltage-gated sodium channels [26,100,101]. Knockdown (*kdr*) resistance has been investigated since it was first observed in a strain of houseflies that survived DDT exposure [102,103,104]. Cases of pyrethroid resistance featuring *kdr*-type mutations have been elucidated, and scientists have found strong associations with point mutations in the *para*-type sodium channel gene [105,106]. Point mutations have also been reported in other species, such as whiteflies, German cockroaches (*Blattella germanica*), and tobacco budworms [107]. Given the scope of the present study, we cannot draw conclusions about the hypothesis that the presence of point mutations results in site-of-action resistance in conjunction with the observed enzyme-mediated metabolic resistance. Nevertheless, unraveling the resistance mechanisms exhibited by this FAW strain is paramount for the re-evaluation and reformulation of current integrated resistance management (IRM) programs in Puerto Rico.

### Implications

It is increasingly clear that areas experiencing recent FAW invasions face a heightened risk of pyrethroid resistance, as suggested by [108]. This risk is compounded by a lack of established knowledge and resources necessary for implementing effective IRM strategies [109,110]. Consequently, immediate and concerted efforts are needed to equip these regions with the tools and expertise required to mitigate the rapid evolution of resistance and safeguard agricultural outputs. By addressing these challenges proactively, we can better prepare these vulnerable regions to manage the threat of FAW globally more effectively.

Puerto Rico is a globally significant location for plant breeding research because of its year-round favorable climate, thereby enabling continuous farming, regulatory frameworks, and a science-friendly environment for biotechnology [25,38,111]. However, these assets also create conditions conducive to high pest pressure, leading to the extensive use of synthetic pest management tools and subsequent resistance development, especially in FAW. To enhance IPM and IRM strategies, IRAC-US and PRABIA [23] have implemented an area-wide resistance management program comprising five work streams: field trials, rotation programs, scouting practices, implementation, and resistance monitoring. Research on pyrethroid resistance in FAW supports the need for these efforts and may contribute to improved IPM practices. Collaborative workshops with the seed industry further aim to address FAW pyrethroid resistance issues [67]. While improving area-wide resistance management programs may take time, such actions are necessary given the broad implications for the global food system posed by pesticide resistance in FAW in Puerto Rico. Climate change may potentially expand the geographical distribution of FAW, establishing conditions conducive to an increased number of generations and progeny, consequently resulting in heightened artificial selection [20].

## Figures and Tables

**Table 1:** Incidents of resistance to pyrethroids in FAW [7].

Compound	Location	Year	Slope	^1^ RR_50_	Source
bifenthrin	USA	1991	2.9	29.4	[40]
	China	2023	2.05	21.8	[41]
cyfluthrin	México	2012	1.04	162.7	[42]
cyhalothrin	USA	1991	1.8	12.5	[40]
cyhalothrin-lambda	^3^ Venezuela	2001	1.31	19.4	[43]
			1.26	41.9	
			1.08	65.7	
			1.23	62	
	Brazil	1998	1.62	12.8	[44]
	México	2008	1.08	204.5	[42]
	Brazil	2008	3.11	28.2	[45]
	Colombia	2010	4.10	34.62	[46]
	Colombia	2010	4.84	50.01	
	China	2021	1.8	31.2	[47]
	^3^ China	2021	0.76	29	[48]
			2.56	317	
			0.58	32	
			0.86	72	
			0.70	26	
	Brazil	2023	2.92	21.5	[49]
cypermethrin	USA	1992	0.8	9.3	[50]
	USA	2006	2.61	10.18	[51]
cypermethrin-zeta	Puerto Rico	2018	1.9	35	[6]
deltamethrin	México	2008	1.04	1002.2	[42]
	Puerto Rico	2018	1.9	25	[6]
	Brazil	2020	1.76	14.23	[52]
	^3^ China	2021	3.21	12	[48]
			3.76	10	
			2.94	12	
			2.31	20	
	China	2023	2.24	13.9	[41]
fenvalerate	USA	1992	2.2	15	[50]
	^3^ China	2021	0.51	15	[48]
			0.98	33	
			1.72	26	
			1.55	11	
fluvalinate	USA	1991	2.9	216	[40]
permethrin	USA	1981	^2^ n/a	17	[53]
	USA	1991	3.3	13.9	[40]
	USA	1992	2	40	[50]
	Mexico	2018	2	19	[6]
	Puerto Rico	2018	1.6	48	
tau-fluvalinate	USA	1992	1.5	263.9	[50]
tralomethrin	USA	1991	5.4	41.2	[40]

^1^—resistance ratio (RR), LC_50_ of resistant strain/LC_50_ of susceptible strain. ^2^—n/a = data not available. ^3^—different locations.

**Table 2:** Concentration–response to esfenvalerate (esfen) and deltamethrin (delta) of fall armyworm from a susceptible lab colony (SUS), a field collection from Ponce Puerto Rico (PPR), and their F_1_ reciprocal crosses (H1 and H2).

Pyrethroid	Strain	n	Slope	SE	^1^ LCs_50_	(95% CI)	^1^ LCs_90_	(95% CI)	^2^ RR_50_	^2^ RR_90_
esfen	PPR	233	1.9	0.4	3.8	(1.3, 6.7)	17	(8.9, 183)	62	123
	SUS	287	3.5	0.6	0.06	(0.04, 0.08)	0.1	(0.09, 0.33)	1	1
	H1 (♂ SUS × ♀ PPR)	369	9.7	1.8	0.8	(0.7, 0.87)	1	(0.97, 1.37)	13	8
	H2 (♀ SUS × ♂ PPR)	424	1.9	0.2	2.2	(1.85, 2.65)	10	(7.9, 14.9)	34	62
delta	PPR	228	3.9	0.41	0.41	(0.35, 0.46)	0.87	(0.74, 1.07)	15	20
	SUS	240	5.8	1.1	0.03	(0.02, 0.03)	0.04	(0.03, 0.10)	1	1
	H1 (♂ SUS × ♀ PPR)	287	3.7	0.4	0.3	(0.26, 0.34)	0.7	(0.58, 0.84)	12	16
	H2 (♀ SUS × ♂ PPR)	335	2.9	0.40	0.398	(0.3, 0.5)	1.11	(0.79, 2.03)	15	25

^1^—LCs_50_ or LCs_90_ (μg/cm^2^). ^2^—Resistance ratio (RR), LC_50_ of resistant strain/LC_50_ of susceptible strain or LC_90_ of resistant strain/LC_90_ of susceptible strain.

**Table 3:** Assessing the mortality rate of FAW strains exposed to esfenvalerate (esfen) with synergists alone and in combination—a comparison of a susceptible lab colony (SUS) and a field collection from Ponce Puerto Rico (PPR).

Pyrethroid	Synergists	Strain	n	Slope	SE	^1^ LCs_50_	(95% CI)	^1^ LCs_90_	(95% CI)	^3^ RR_50_	^2^ SR_50_	^2^ SR_90_
esfen	-	PPR	233	1.9	0.4	3.76	(1.3, 6.7)	17.2	(8.9, 183)	62	-	-
	PBO		327	1.7	0.3	1.07	(0.6, 1.7)	6.0	(3.3, 19)	18	3.5	2.9
	DEM		528	1.8	0.4	0.49	(0.2, 0.76)	2.4	(1.4, 8)	8	7.7	7.1
	DEF		384	1.8	0.1	0.53	(0.4, 0.66)	2.8	(2.1, 4)	9	7.1	6.1
	VER		432	3.0	0.6	0.80	(0.52, 1.2)	2.1	(1.3, 5.3)	13	4.7	8.1
	PBO + DEM + DEF + VER		335	1.9	0.3	0.31	(0.2, 0.45)	1.4	(0.9, 3)	5	12	12
	-	SUS	287	3.5	0.6	0.06	(0.04, 0.08)	0.1	(0.09, 0.33)	1	-	-
	PBO		288	2.6	0.3	0.01	(0.011, 0.015)	0.04	(0.03, 0.06)	0	4.6	3.3
	DEM		432	2.5	1.2	0.33	* -	1.1	* -	5	0.2	0.1
	DEF		240	3.1	0.3	0.04	(0.036, 0.05)	0.1	(0.09, 0.15)	1	1.4	1.3
	VER		479	3.5	0.3	0.12	(0.1, 0.13)	0.3	(0.23, 0.32)	2	0.5	0.5
	PBO + DEM + DEF + VER		430	4.1	1.6	0.07	(0.032, 0.28)	0.1	(0.08, 245)	1	0.9	1.0

^1^—LCs_50_ or LCs_90_ (μg/cm^2^). ^2^—Synergist ratio (SR) = LC_50_ of esfenvalerate without synergist/LC_50_ of esfenvalerate + synergist. d.f. = degrees of freedom. ^3^ Resistance ratio (RR), LC_50_ of resistant strain/LC_50_ of susceptible strain. PBO = piperonyl butoxide; DEM = diethyl maleate; DEF = S,S,S-tributyl phosphorotrithioate; VER = (±)-verapamil hydrochloride. *—No confidence intervals could be calculated.

**Table 4:** Assessing the mortality rate of FAW strains exposed to deltamethrin (delta) with synergists—a comparison of a susceptible lab colony (SUS) and a field collection from Ponce Puerto Rico (PPR).

Pyrethroid	Synergists	Strain	n	Slope	SE	^1^ LCs_50_	(95% CI)	^1^ LCs_90_	(95% CI)	^3^ RR_50_	^2^ SR_50_	^2^ SR_90_
delta	-	PPR	228	3.9	0.4	0.406	(0.3, 0.4)	0.9	(0.74, 1.07)	15	-	-
	PBO		239	2.0	0.5	0.184	(0.04, 0.44)	0.8	(0.3, 55)	7	2	1
	DEM		239	2.4	0.3	0.207	(0.16, 0.24)	0.7	(0.54, 0.97)	7.8	2	1
	DEF		335	1.3	0.2	0.024	(0.008, 0.05)	0.3	(0.1, 1.2)	0.92	17	3
	VER		335	2.3	0.4	0.091	(0.05, 0.17)	0.3	(0.17, 1.61)	3.4	4	3
	-	SUS	240	5.8	1.1	0.026	(0.021, 0.034)	0.04	(0.03, 0.106)	1	-	-
	PBO		283	2.6	0.4	0.009	(0.005, 0.012)	0.03	(0.017, 0.07)	0.34	3	1.6
	DEM		336	3.4	0.5	0.026	(0.02, 0.03)	0.06	(0.045. 0.11)	1	1	0.7
	DEF		239	2.3	0.4	0.02	(0.015, 0.023)	0.07	(0.05, 0.14)	0.76	1.3	0.6
	VER		528	2.7	0.3	0.028	(0.02, 0.03)	0.08	(0.063, 0.12)	1.05	1	0.5

^1^—LCs_50_ or LCs_90_ (μg/cm^2^). ^2^—Synergist ratio (SR) = LC_50_ of deltamethrin without synergist/LC_50_ of deltamethrin + synergist. ^3^—Resistance ratio (RR), LC_50_ of resistant strain/LC_50_ of susceptible strain. PBO = piperonyl butoxide; DEM = diethyl maleate; DEF = S,S,S-tributyl phosphorotrithioate; VER = (±)-verapamil hydrochloride

## Data Availability

The data presented in this study is available in the article.
